# Effects of Season on Donor and Recipient Cows and Calf Performance from Birth to Weaning in Embryo Transfer Programs in the Tropics

**DOI:** 10.3390/ani11123596

**Published:** 2021-12-19

**Authors:** José Francisco Martínez, Carlos Salvador Galina, Pablo Ortiz, Martín Guillermo Maquivar, Juan José Romero-Zúñiga

**Affiliations:** 1 Department of Reproduction, Faculty of Veterinary Medicine and Zootechnics, National Autonomous University of Mexico, Mexico City 04510, Mexico; jfran.malba@gmail.com (J.F.M.); cgalina@unam.mx (C.S.G.); ortizmontufar.pablo@gmail.com (P.O.); 2 Department of Animal Sciences, Washington State University, Pullman, WA 99164, USA; martin.maquivar@wsu.edu; 3 Population Medicine Research Program, School of Veterinary Medicine, Universidad Nacional, Heredia 40101, Costa Rica

**Keywords:** embryo transfer, calf growth, season, tropics, *Bos indicus*

## Abstract

**Simple Summary:**

It is well-known that embryo transfer is a powerful tool which can increase the number of offspring of donor cows, thus improving cattle efficiency. The recipient dams are vital in a successful embryo transfer program, as they will sustain the length of the pregnancy and directly impact the development of the embryo and the productive performance of the animal after birth. Evaluating the effect of season on donors, recipients and delivered offspring from a private farm, we found that the seasonal effect is more apparent in donor and calf performance than in the recipients. It seems to be that the performance of the calves measured by birth and weaning weight favors the embryo-transferred offspring. However, there is a bias towards measuring the performance of calves born by embryo transfer as their condition was favorable compared to calves born by natural mating, as the latter calved at pasture without supervision, whereas the former the recipient dams calved in selected pastures.

**Abstract:**

The aim of this study was to assess the seasonal effect of an embryo transfer program in the tropics on the donor response, recipient reproductive performance and calf growth from birth to weaning. This study included five-year records from 145 donors, 1149 embryo transfers (ET) and 609 in calves. The effect of the season (dry or wet) was evaluated at the time of embryo flushing, embryo transfer and birth of the calves. There was a seasonal effect on the yield and quality of the embryos. The number of nonfertilized and transferable good quality embryos increased in the wet season. For the recipients, the probability of pregnancy after an ET decreased by 6% for each year of the dam’s age. However, no seasonal effect was found when comparing ET calves with their control group (natural mating—NM), nevertheless, weaning weight was associated with birth body weight, treatment, sex of the calf, season at birth, year of treatment, and dam’s age. Calves born by NM had lower average daily gain (ADG), and male calves registered higher gains than females. Likewise, calves born during the rainy season had lower ADG compared with calves born during the dry season. In conclusion, this study shows that seasonal effect is more apparent in donor and calf performance than in the recipients.

## 1. Introduction

Several studies have reported a strong seasonal effect on embryo production yields and quality of the embryos in *Bos indicus* cattle [1,2] due to high variation in climatic conditions and pasture availability. Embryo quality differences among seasons is more apparent when microscopical assessment is undertaken. For example, Marquez et al. [3], found essential differences in the number of apoptotic cells in embryos produced in the wet season compared with the dry season in the Mexican tropics. Bényei et al. [4] in Brazil, recommended that to achieve higher conception rates, the production of embryos should be carried out during the wet and cooler season and transferred later during the dry and hot season. Paradoxically, Nasser et al. [5] in Brazil, in an extensive study carried out in *Bos taurus* × *Bos indicus* beef recipients, reported that season affected conception rate following the transfer of fresh in vitro produced (IVP) embryos. Lower pregnancy rates were observed during the autumn and winter compared with the spring and summer months (41.1% (448/1090) vs. 48.1% (1760/3658), respectively). These results are probably related to the dry weather and the poorer availability/quality of forage during the autumn/winter period observed in tropical grasses. However, the effect of variations in the temperature humidity index (THI) also has to be considered, as pointed out in recent studies by our group [6].

The two distinct seasons in the tropics vary according to the geographical location of countries and topographical regions, so a dry or wet season could have different interpretations among researchers working in tropical conditions. Nonetheless, one interesting feature of the embryo transfer industry relates to the vast amount of information concerning the array of hormonal treatment schemes used to maximize the production yield of good-quality embryos. However, scarce information is available on the combined effect of season and the condition of the recipient animals on successful embryo transfer programs. Moreover, the recipient animals will sustain the length of the pregnancy and directly impact the development of the embryo and the productive performance of the animal after birth [7]. To our knowledge, there are no studies assessing the impact on growth of the embryo-transferred calf and the time of birth. Therefore, the objectives of the present study were to evaluate the effect of the season on (1) reproductive response of the donor cows subjected to an embryo recovery program, (2) reproductive performance of the recipient, and (3) growth performance from birth to weaning of the calves born by embryo transfer.

## 2. Materials and Methods

### 2.1. Location

A retrospective five-year study (2014–2019) was undertaken in a private farm producing purebred Brahman cattle in the municipality of Tamiahua, Veracruz, Mexico located at 20°57′18″ latitude north and 97°23′58″ latitude west, with an altitude of 14 m above sea level. The region is characterized by a tropical climate with 24.9 °C annual average temperature and 1241 mm of rainfall. This farm has been using embryo transfer for the last 15 years.

The nutritional management in this farm was mainly based on intensive rotational grazing of *Cynodon nlemfuensis* (African star grass), *Paspalum spp*. and *Axonopus pp* pastures, supplemented with minerals and water ad libitum. Traditionally, cattle have been immunized against brucellosis (*Brucella abortus* strain 19 vaccine, 3 to 9 months old), clostridial polyvalent vaccine (twice a year, total herd), leptospirosis polyvalent vaccine (twice a year, total herd), a polyvalent vaccine for infectious bovine rhinotracheitis, bovine viral diarrhea, bovine parainfluenza type 3 and bovine respiratory syncytial virus (all the herd, cows and heifers before breeding) and paralytic rabies (all the herd once a year and in calves at 3 and 9 months old).

### 2.2. Data Selection

A total of 1659 records, 1149 embryo transfers (ET) and 510 from natural mating (NM) were considered for this study. Three separate analyses were performed to assess the objectives. First, donor cows were classified based on the season when they were subjected to a superovulatory hormonal treatment for *Bos indicus* cattle according to Baruselli et al. [8]. Two 0.5 mL straws of semen per cow were artificially inseminated from bulls born in the farm and varied by season and year as a part of a genetic improvement program. Therefore, this factor was not included in the model. The straws were prepared in a well-established private company in the state of Querétaro, Mexico (Mexitube Germany) where the bulls remain for as long as is required to produce doses with a sperm concentration of 20 × 10^6^ sperm count/straw. Seven days after artificial insemination, cows were flushed with ViGRO_TM_ complete flush solution (Bioniche Animal Health, Ontario, CA, Canada) and the embryos were collected in a filter (EmCon, Bioniche Agtech, WA, USA) and subsequently taken to the laboratory. Embryos and cells were searched for in a Petri dish with a stereo microscope (Stereo Zoom^®^6, Carl Zeiss, Hombrechtikon, Switzerland). Furthermore, embryos and cells obtained by flushing were classified as: transferable, nonfertilized oocytes, or degenerative cells [9]. The treatment regime was the same throughout the study to obtain embryos.

The second level of the analysis included recipients that were synchronized with a fixed-time protocol and consisted of 2 mg of estradiol benzoate (EB) (Gonadiol^®^, Zoetis, Mexico City, Mexico) intramuscularly and a CIDR^®^ implant (1.9 g of progesterone; Zoetis, Mexico City, Mexico) on day 0. At day 8, the CIDR was removed, and each cow was injected intramuscularly with 1 mg of EB, 25 mg of dinoprost tromethamine (Prostaglandin _F2α_, Lutalyse^®^, Zoetis, Mexico City, Mexico) and 400 IU of eCG (Novormon 5000^®^, Zoetis, Mexico City, México). The embryos were transferred fresh from the donors (after being flushed and classified) to the recipients that have a visible corpus luteum under transrectal ultrasonography at day 18. The reproductive response was assessed in the recipients by calculating the percentage of reabsorptions (≤45 days), abortions (>45 days), as well as confirmed pregnant and nonpregnant cows after an ET program by transrectal ultrasonography using a 7.5 MHz transductor (Aloka SSD 500, Tokyo, Japan) performed on days 30 and 60 after being transferred.

Finally, the third level of analysis involved 609 records from calves produced by ET, and as a control group, 510 records from NM. The bulls used for natural mating were also born in the farm, subjected to a prior breeding soundness evaluation and were part of the genetic improvement program. Males varied annually and seasonally according to the farm program. The birth and weaning weights were adjusted by the age of the mother to decrease the error between calves and used to calculate the average daily gain (see Table 1). To assess the effect of the season, birth and weaning weights and average daily gain were analyzed for ET and NM.

### 2.3. Statistical Analysis

#### 2.3.1. Donor Females

A total of 145 donor Brahman cows fit for superovulation were used in the analysis. Their age ranged between 4–11 years and their body condition score from 4–6 in a scale of 1 to 9 (where 1 = emaciated, 9 = obese) [10]. After flushing, the final classification of the embryos obtained from superovulation treatment was used as the dependent variable of the study: transferable (TRANS), nonfertilized oocytes (N-FERT), and degenerative cells (DEG). Transferable embryos were classified on a scale from 1 to 3 (where 1 = excellent and 3 = poor) [11]. Complementarily, cow’s age, season (rainy and dry), and year in which the donors were subjected to the hormonal treatment, were the independent variables of the model. Monthly rainfall expressed in millimeters (mm), in Tuxpan de Rodríguez Cano (Veracruz), Mexico, was used to classify the months between May to October as the rainy season (average 164 mm, 16–27° Celsius), and November to April as the dry season (average 54 mm, 11–22° Celsius) [12].

The number of TRANS, N-FERT, DEG embryos were taken as a continuous variable. A Shapiro–Wilk test for each category was performed to test for normality. Descriptive statistics of central tendency, dispersion, and position (mean, standard deviation, quartiles, minimum, and maximum) were calculated for each independent variable. Finally, generalized linear models adjusted by the correspondent distribution and link function were performed to assess the effect of the independent variables on the dependent variables. The lowest value of the Akaike Information Criterion (AIC) was used to determine the optimal specification of the regression and select the parameterized model with higher efficiency. All the analyses were carried out in JAMOVI (version 1.6.23, Sydney, Australia) [13].

#### 2.3.2. Embryo Transfer Recipients

The animals selected to become recipients were Brahman or crossbreeds, ranged between 2–11 years of age, were cycling and had a body condition score ranging from 4 to 6 on a scale of 1 to 9, as previously stated [10]. This level of the analysis included data from 1149 embryo transfer recipients. A frequency distribution of the services was performed according to breed and cow age, and also by year and season in which the transfer of the embryo occurred. A detailed analysis of fertility was carried out, first, the global and specific frequency (absolute and relative) for the ET outcome: reabsorption, abortion, pregnant or nonpregnant, and then the difference between proportions was tested through a chi-squared test using a significance threshold α of 0.05.

Additionally, the probability of an effective outcome, defined as a confirmed pregnancy, regarding the age and breed of the dam, season and year of service, and quality (1–3) of the embryo, was calculated using a nonconditional logistic binomial regression. For this purpose, the outcome of the ET was recorded as pregnant and nonpregnant cows. The logistic procedure consisted of two steps: (1) univariate analysis (2) multivariate analysis. In the second step, all variables with *p* < 0.25 in the univariate analysis were included. Then, a backward building model was followed based on the likelihood ratio test. The process of exclusion–inclusion of each variable into the multivariate model tested confounding and interaction by comparing the estimated coefficients in the new model with the coefficients and likelihood ratio of the old model. Confounding effect was deemed present if at least one coefficient changed by more than 0.1 (if the coefficient had a value between −0.4 and 0.4) or if at least one coefficient changed by more than 25% (if the coefficient ranges between < −0.4 or >0.4). Finally, variables excluded in the univariate step were checked for collinearity with the variables in the final model to check for potential confounding by calculating simple correlations. All analyses were carried out in JAMOVI (version 1.6.23, Sydney, Australia) [13].

#### 2.3.3. Calves

This level of analysis involved 609 records from Brahman calves produced by ET and as a control group, and a total of 510 records were from NM. The variables chosen in the model were the effect of the treatments (NM, ET), season at birth (dry or rainy), year of treatment (from 2013 to 2018), sex of the calf (male or female), the age of the recipient, at-birth body weight (BBW) and weaning body weight (WBW). Data collected for calves at birth (season and body weight) and weaning weight were adjusted by the age of the dam on all the calves. The BBW was corrected (Table 1) based on the age of the dam [14], and the WBW was adjusted at 205 days using the following formula:

Adjusted 205-day WBW = ((weaning weight [Kg]) − (Birth weight [Kg])/weaning age × 205) + birth weight [Kg] + age of dam adjusted factor.(1)

**Table 1:** The adjustment factors used in the present study.

Age of the Dam	Birth Weight (Kg) Adjustment Factor	Male Weaning Weight (Kg) Adjustment Factor	Female Weaning Weight (Kg) Adjustment Factor
2	+3.62	+27.21	+24.49
3	+2.26	+18.14	+16.32
4	+0.90	+9.07	+8.16
5–10	0	0	0
≥11	+1.36	+9.07	+8.16

Source: [14].

The average daily gain (ADG) was calculated using the formula:

ADG (Kg/d) = (Adjusted Weaning weight − adjusted birth weight)/weaning age (expressed in days).(2)

A Shapiro–Wilk normality test for each dependent variable (BBW, WBW, and ADG) was performed. In addition, descriptive statistics of central tendency, dispersion, and position (mean, standard deviation, quartiles, minimum, and maximum) were calculated to BBW and WBW through each independent variable. A generalized linear model (GLM) according to the distribution of the dependent variables (BBW, WBW, and ADG) with their respective link function was used to test the main effects and the interactions of the independent variables. A forward stepwise modeling method was performed if an independent variable was found not to be significant at *p* < 0.10 but still was maintained temporarily in the model. Only the variables with a *p* < 0.05 remained in the model. The lowest AIC and the R-squared of the model were used as criteria to select the best fitting model. Analyses were carried out using JAMOVI (version 1.6.23, Sydney, Australia) [13].

## 3. Results

### 3.1. Donor Females

**Figure 1:**
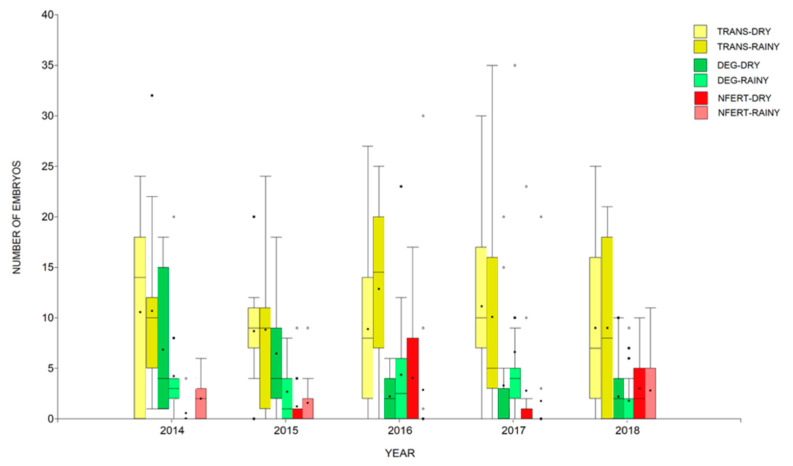
Distribution of the embryo flushing products (transferable—TRANS; nonfertilized oocytes—NFERT; degenerative cells—DEG) after a superovulation process, by year and season of the treatment. The box area indicates the interquartile range, median and mean are shown with a line and dot, respectively, and dots rather than the bars represent the outlier data.

The number of embryos collected after each cycle of flushing were not normally distributed (W > 0.06; *p* < 0.0001). Most of them were classified as TRANS (mean = 9.83, median = 9.0; interquartile range: 3.0–15.0), followed by DEG embryos (mean = 3.68, median = 2; interquartile range: 0–4), and N-FERT were the least frequent ones (mean = 2.48, median = 0.0; interquartile range: 0.0–3.0). The frequencies of TRANS, DEG and N-FERT varied by year and season (Figure 1).

The number of embryos classified as TRANS were reduced for each year of the age of the donor (*p* = 0.002), whilst the year and the season of treatment, showed a significant interaction with the age of the donor (*p* < 0.001). However, this interaction may be overestimated due to the effect of age on season and year of the study. Nevertheless, the interaction between age and season shows a trend of producing more transferable embryos in the rainy season. Similar results were observed for DEG cells and N-FERT. A strong association was seen when all variables interacted (Table 2).

### 3.2. Embryo Transfer Recipients

**Figure 2:**
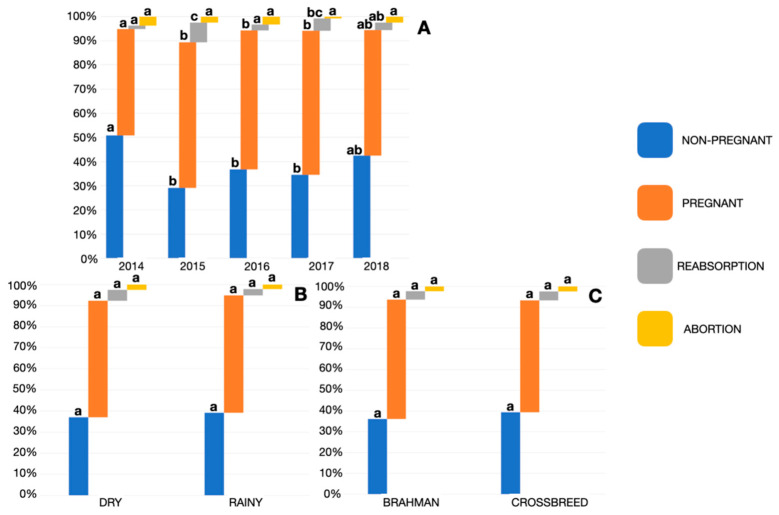
Embryo transfer outcome frequencies by year of service (**A**), season (**B**), and breed of the recipient cow (**C**). Different letters between nonpregnant, pregnant, reabsorption and abortion columns were significantly different *p* < 0.05.

According to the year of treatment, there were significant differences in the percentage of pregnant and nonpregnant cows (*p* < 0.05). This finding, partially due to the number of abortions was low (<3.5% in all years), also, the numbers of embryonic reabsorptions were significantly different only in two years: 2015 (8.3%) and 2017 (5.1%). However, when the frequencies of the ET outcome were analyzed according to breed and season of the year there were no significant differences (*p* > 0.05) in any category (Figure 2).

The probability of not achieving a pregnancy increased by 6% for each year of the recipients age (*p* = 0.01), the probability of achieving a pregnancy was equal for quality 1 and 2 embryos (*p* = 0.71), and the risk of not achieving a pregnancy increased to almost double when analyzing category 3 embryos (*p* = 0.01) with respect to category 1 (OR = 1.88; 95%CI: 1.17–3.02). The probability of becoming pregnant is significantly modified as an effect of the year in which the transfer is performed (*p* < 0.001) (Table 3).

### 3.3. Calves

As shown in Table 4, BBW was associated with treatment, sex of the calf, season at birth, year of treatment, and dam’s age (*p* < 0.001). Calves born by NM had lower BBW when compared to those born by ET (*p* = 0.003). Male calves recorded higher birth weights than females (*p* < 0.001). During the rainy season, BBW was lower than in the dry season (*p* < 0.001). Only 2018 showed differences in the BBW compared to 2013 (*p* < 0.001), although in the raw value, disparities around 3 kg were observed between the year 2018 and years 2014 to 2016. Dam’s ages 4–5 (*p* < 0.005), as well as 6–7 and 8–9, had higher BBW compared to ages 2–3 (*p* < 0.001). Regardless of the effect of treatment, there were no interactions between the variables studied.

Weaning weight was associated with the BBW, treatment, sex of the calf, season at birth, year of treatment, and dam’s age (*p* < 0.001). The WBW increases with BBW (*p* = 0.002). Calves born by NM had a lower weight when compared to those born through ET (*p* < 0.001). Males recorded higher WBW than females (*p* < 0.001). Calves born during the rainy season had lower WBW than those born during the dry season (*p* < 0.001). Calves from dams with ages ranging 6–7 and 8–9 years recorded higher weaning weights compared to those from cows aged 2–3 years (*p* < 0.001), while those aged ≥10 years showed a tendency to have a lower WBW (*p* = 0.066). The years 2014 and 2015 recorded higher WBW compared to 2013 (*p* < 0.001), while 2017 showed the lowest weights (*p* < 0.001) (Table 5).

The average daily weight gain (ADG) was statistically associated with treatment, calf sex, season at birth, year of treatment, and dam’s age (*p* < 0.001). Thus, calves born by NM had lower daily weight gain (*p* < 0.001), and male calves registered higher weight gain than females (*p* < 0.001). Likewise, calves born during the rainy season had lower daily weight gain than calves born during the dry season (*p* < 0.001). Calves born in 2014 had higher gain than those born in 2013 (*p* = 0.029), while those born in 2017 showed lower weight gain (*p* < 0.001). Except for cows aged ≥10 years (*p* = 0.615), all other ages showed higher ADG than those aged 2–3 years (*p* < 0.001) (Table 6).

## 4. Discussion

One consideration often omitted by the ET industry is that the recipient animal host nurtures and provides adequate conditions for the development of a foreign embryo for the length of the pregnancy. The donor cow is involved only during the early embryonic development (5–7 days) before it is flushed and then transferred. Research is therefore needed on the dynamic physiological interactions of the embryo, and later fetus, in the recipient reproductive tract. Similarly, the fetal programing that occurs during gestation and the genetic effect on the calves conceived by ET requires clarification [15].

### 4.1. Donor Females

The average number of embryos for transferring to recipient cows was on average 9.8 per superovulatory cycle. Whereas the number of nontransferable embryos (degenerative or nonfertilized) amounted to 3.68 and 2.48, respectively, with age being a main factor affecting the number of nonviable embryos. This figure agrees with Naranjo-Chacón et al. [9] in their study on *Bos indicus*. As probably anticipated from previous research on the superovulatory regimen, we found an effect of the year of the study and the season. This agrees with previous studies on Holstein cattle [16], which have shown that the number of nonfertilized and transferable embryos increased in the cooler part of the year and that degenerative embryos are not affected by season. Even in Gyr cows, well-known for their adaptation to tropical climates, Torres-Júnior et al. [17], documented a damaging effect of heat stress in the in vitro blastocyst production compared to cooler conditions. Additionally, under field conditions, the embryos collected from a superovulatory cycle are generally classified by subjective measures. Therefore, it is important to note that just because an embryo was classified as transferable does not mean it will result in a pregnancy due to possible cellular damage [18]. Moreover, the inability of the recipient to sustain pregnancy, due to asynchrony of the morphological and endocrinological interactions between the embryo and the endometrium, results in an unsuccessful establishment of the pregnancy [19]. In the current experiment, a relatively small number of embryos classified as nontransferable and degenerated does give an indication that perhaps the personnel evaluating the embryos probably overestimated the grading of the cells classified as viable for transfer [20]. Additionally, the effect of breed is an important aspect in ET programs. In effect, Watanabe et al. [21], in a large field study in Brazil, found that Nelore cattle had fewer oocytes recovered per ovum pick-up compared to Senepol and Holstein. Early reports, such as those of Barros and Nogueira [22] and Barusselli et al. [23] in *Bos indicus*, have indicated differences in follicular wave synchronization, ovulatory and fertilization rates, in response to superovulatory treatments compared to *Bos taurus* cattle. Another important aspect relates to the selection of recipients, which in general terms, is under appreciated and plays an important role in a successful embryo transfer program [15]. Nutritional status and health of the female, response to the estrus synchronization regimen, and the timing of ET are factors that can affect the success of the program.

Other factors such as climatic conditions under which *Bos indicus* cattle are raised, high humidity and heat stress also influence the success of reproductive biotechnologies in beef dams, reducing fertilization rate and embryo quality, while increasing the rate of pregnancy loss [19]. Consequently, this seasonal effect has been shown to affect cellular and physiological responses in the animals. Marquez et al. [3], suggested that embryos collected during the dry season show more cellular damage (increased number of apoptotic blastomeres) compared to embryos cryopreserved in the rainy season, which appeared morphologically better equipped to result in a pregnancy following ET. The present study showed a strong seasonal effect on the yield and quality of the embryos, which can be partially explained by climatic changes. In recent years, global temperature has increased, resulting in differences in reproductive responses. This phenomenon has been observed in Holstein cattle exposed to high temperature and humidity. Pavani et al. [24] demonstrated that cattle exposed to a 70.6 THI index showed a significant decrease in conception rate, cleavage and embryo development, resulting in less-fertile animals.

### 4.2. Embryo Transfer Recipients

At odds with previous research, abortion or possibly reabsorption rates could amount to between 10 and 12% in recipient cows between 38 and 90 days post ET in temperate areas [25,26,27]. Bényei et al. [4] in Brazil, found that pregnancy losses were around 10%. Our findings point out to a low number of abortions (<3.5% across all years), and the number of embryonic reabsorptions was significantly high only in two years: 2015 (8.3%) and 2017 (5.1%). It is difficult to explain the low number of abortions in the present study; perhaps adequate sanitary measurements contributed to this low percentage. The nature of the study does not allow us to estimate the quality of the health herd management. Moreover, even when the frequencies of the outcome of the ET were analyzed according to breed and season of the year, there were no significant differences in any category. Baruselli et al. [8], compared pregnancy losses between ET and AI (artificial insemination) in repeat-breeder cows, and concluded that there were no differences in fertility among these groups, reinforcing the hypothesis that the infertility problem of repeat-breeders, or any cows, may be associated with oocyte quality and/or failure of early embryo development in the female reproductive tract.

Additionally, results from the present study showed that the probability of not achieving a pregnancy after an ET program increases by 6% for each year of the dams age, the probability to become pregnant was equal for quality 1 and 2 embryos, and the risk of not achieving a pregnancy increased to almost double when analyzing quality 3 embryos. Related studies such as the one of Roper et al. [28], showed that the age of the female and quality of the embryo play an important role in attaining pregnancy. Again, in keeping with our results, embryo quality tended to affect pregnancy rates with the primary difference being between 1 and 3. Perhaps the careful selection of recipient animals in the present study could also have minimized the risk of failing to become nonpregnant animals.

### 4.3. Calves

Calves born by NM showed lower BBW than those born by ET. Likewise, Pimenta-Oliveira et al. [29], found differences in birth weight of the offspring between the IVP embryos and AI. Nonetheless, Lopes et al. [30], compared the growth of calves born by ET with those from AI, and their results showed much similarity between groups in terms of pregnancy rates, gestation length and calves’ birth weight. In our study, male calves recorded higher birth weights than females. In a similar study, Camargo et al. [31], found gestation length was similar between ET and AI groups, but ET-derived offspring were heavier than AI, especially male calves. The oddity of these differences could be the consequence of different conditions of the cows calving at pasture, or perhaps there are true differences in calf performance. More research is needed on this issue. During the rainy season, BBW was lower than in the dry season, where there was an effect related to the year of the study. Similarly, Hernández-Hernández et al. [32], have reported that year-to-year variation is one of the main factors affecting the weight of the calves’ repeatability in long-term studies.

As anticipated, weaning weight was associated with the BBW, treatment, sex of the calf, season of birth, year of treatment, and dam’s age. The WBW increased with heavier calves at birth. Calves born from NM had a lower weaning weight than those born by ET. Wilson et al. [33], demonstrated that the average weight of ET calves was not different from the average weight of AI/NM calves at 205 days. The disparity of this comparison can be the consequence of a compensatory factor affecting early weight of the calves not reflected at weaning. In effect, Segura et al. [34], found that male calves weighed more at birth and weaning than females, but there was a year x season interaction, indicating that the latter affects the performance of calves or cows.

As with BBW and WBW, the average daily weight gain (ADG: NM 0.72; ET 0.92) was statistically associated with all the study variables. Effectively, Pimenta-Oliveira et al. [29] found no differences in ADG between ET and AI. This effect could be due to a similar selection process for dams and bulls in both reproductive techniques, thus not affecting the weight of the offspring until weaning. Funston et al. [35] indicated that heifer birth weight was lower for heifers born early in the breeding season. The ADG tended to be significantly less efficient for heifers born in the first calving period, illustrating the fact that timing of birth has a prominent effect on their performance as adults.

## 5. Conclusions

The number of embryos classified as transferable were not different with respect to the season in which the superovulatory treatment was applied. Nonetheless, the number of nontransferable embryos increased in the dry season. Cows used as recipients had similar performance in resorptions, abortions and pregnancy over the years and seasons; likewise, their calves performed similarly to those born by natural mating. Apparently, the effect of season is less important for embryo production and pregnancy success in recipients than for calf growth, where this factor was relevant.

## Figures and Tables

**Table 2:** Results of the generalized linear model for the categories, using a Poisson distribution with a log link function.

Embryo Category	Model Info	Variable	*p*
Transferable	R-squared = 0.0801	Cow’s Age (AGE)	0.002
	AIC = 1514.233	Year of treatment (YEAR)	0.037
	Deviance = 966.331	Season of treatment (SEASON)	0.496
	Residual DF = 122	YEAR ✻ AGE	<0.001
	Chi-squared/DF = 7.003	AGE ✻ SEASON	<0.001
Degenerative	R-squared = 0.233	Cow’s Age (AGE)	<0.001
Cells	AIC = 1002.653	Year of treatment (YEAR)	<0.001
	Deviance = 647.571	Season of treatment (SEASON)	0.114
	Residual DF = 122	AGE ✻ YEAR	<0.001
	Chi-squared/DF = 5.394	AGE ✻ SEASON	<0.001
Nonfertilized	R-squared = 0.383	Cow’s Age (AGE)	0.505
Oocytes	AIC = 819.803	Year of treatment (YEAR)	0.101
	Deviance = 550.812	Season of treatment (SEASON)	0.047
	Residual DF = 109	AGE ✻ YEAR	<0.001
	Chi-squared/DF = 5.366	YEAR ✻ SEASON	0.017
		AGE ✻ YEAR ✻ SEASON	<0.001

✻ Denotes interaction between the variables.

**Table 3:** Best fit nonconditional logistic regression model for risk of being nonpregnant in embryo transfer recipients (ET). Only associated variables are shown, even though all variables and interactions were tested.

Predictor	*p*	OR	95% CI		Omnibus Likelihood Ratio Tests		
			Lower	Upper	χ²	DF	*p*
Intercept	0.36	0.82	0.54	1.25	7.64	1	0.006
Cow’s age	0.01	1.06	1.02	1.11			
Embryo quality							
2	0.71	1.06	0.78	1.43	6.75	2	0.034
3	0.01	1.88	1.17	3.02			
Year of treatment							
2015	<0.001	0.38	0.24	0.61	21.10	4	<0.001
2016	0.00	0.52	0.34	0.81			
2017	<0.001	0.47	0.30	0.72			
2018	0.07	0.67	0.44	1.03			

Reference levels: Embryo quality 1; Year of treatment: 2014; Cow’s age: as a continuous variable from 2 years old. Degrees of freedom (DF).

**Table 4:** Best fit model for fixed effects on birth body weight (BBW) in kilograms by a generalized linear model procedure using a gamma distribution with a log link function.

Variable	Level	EMM	*p*	Log Likelihood Ratio Tests		
				X²	DF	*p*
Intercept	Intercept		<0.001			
Treatment (TREAT)	NM	38.2	0.003	8.92	1	0.003
Calf sex (SEX)	Male	39.1	<0.001	34.22	1	<0.001
Season at birth (SEASON)	Rainy	38.9	<0.001	18.45	1	<0.001
Year of treatment (YEAR)	2014	39.4	0.186	172.67	5	<0.001
	2015	39.1	0.531			
	2016	39.6	0.084			
	2017	38.2	0.091			
	2018	36.1	<0.001			
Cow’s age (AGE)	4–5	38.5	0.005	34.33	4	<0.001
	6–7	38.8	<0.001			
	8–9	39.4	<0.001			
	≥10	38.3	0.094			

Model info: R-squared = 0.180; AIC = 5757.398; Deviance = 7.867; Residual DF = 1095; Chi-squared/DF = 0.0064. Reference level [EMM]: TREAT (ET [38.9]); SEX (female [38.0]); SEASON (dry [39.0]); AGE (2–3 [38.3]), YEAR (2013 [38.9]). Estimated marginal means (EMM), degrees of freedom (DF), natural mating (NM), embryo transfer (ET).

**Table 5:** Best fit model for fixed effects on weaning body weight (WBW) in kilograms by a generalized linear model procedure using a normal distribution with an identity link function.

Variable	Level	EMM	*p*	Log Likelihood Ratio Tests		
				X²	DF	*p*
Intercept	Intercept		<0.001			
Birth Body Weight (BBW)	BBW	232.1	0.002	9.22	1	0.002
Treatment (TREAT)	NM	211.3	<0.001	45.58	1	<0.001
Calf sex (SEX)	Male	240.0	<0.001	16.72	4	0.002
Season at birth (SEASON)	Rainy	223.6	<0.001	53.13	1	<0.001
Cow’s age (AGE)	4–5	235.5	0.508	295.18	1	<0.001
	6–7	239.1	0.021			
	8–9	234.9	0.002			
	≥10	225.4	0.066			
Year of treatment (YEAR)	2014	248.2	0.002	77.91	5	<0.001
	2015	242.0	0.07			
	2016	229.7	0.505			
	2017	217.3	<0.001			
	2018	226.6	0.172			

Model info: R-squared = 0.398; AIC = 10,337.72; Deviance = 1,310,000; Residual DF = 1005; Chi-squared/DF = 1303.526. Reference level [EMM]: TREAT (ET [254]); SEX (female [224]); SEASON (dry [241]); AGE (2–3 [228]), YEAR (2013 [233]). Estimated marginal means (EMM), degrees of freedom (DF), natural mating (NM), embryo transfer (ET).

**Table 6:** Best fit model for fixed effects on average daily weight gain (ADG) in kilograms by a generalized linear model procedure using a normal distribution with an identity link function.

Variable	Level	EMM	*p*	Log Likelihood Ratio Tests		
				X²	DF	*p*
Intercept	Intercept		<0.001			
Treatment (TREAT)	NM	0.72	<0.001	351.7	1	<0.001
Calf sex (SEX)	Male	0.85	<0.001	46	1	<0.001
Season at birth (SEASON)	Rainy	0.79	<0.001	33.2	1	<0.001
Year of treatment (YEAR)	2014	0.88	0.029	68.8	5	<0.001
	2015	0.83	0.674			
	2016	0.81	0.214			
	2017	0.75	<0.001			
	2018	0.81	0.231			
Cow’s age (AGE)	4–5	0.83	<0.001	31.9	4	<0.001
	6–7	0.85	<0.001			
	8–9	0.85	<0.001			
	≥10	0.79	0.615			

Model info: R-squared = 0.331; AIC = −853.172; Deviance = 25.634; Residual DF = 1017; Chi-squared/DF = 0.0252. Reference level [EMM]: TREAT (ET [0.92]); SEX (female [0.79]); SEASON (dry [0.85]); AGE (2–3 [0.78]), YEAR (2013 [0.84]). Estimated marginal means (EMM), degrees of freedom (DF), natural mating (NM), embryo transfer (ET).

## Data Availability

All data generated or analyzed during this study are included in this published article.
